# The Toll-Like Receptor 4 Polymorphism Asp299Gly but Not Thr399Ile Influences TLR4 Signaling and Function

**DOI:** 10.1371/journal.pone.0093550

**Published:** 2014-04-02

**Authors:** Huaicong Long, Brian P. O'Connor, Rachel L. Zemans, Xiaofang Zhou, Ivana V. Yang, David A. Schwartz

**Affiliations:** 1 Center for Genes, Environment and Health, National Jewish Health, Denver, Colorado, United States of America; 2 Department of Geriatrics, Sichuan Academy of Medical Sciences & Sichuan Provincial People's Hospital, Chengdu, Sichuan, P.R. of China; 3 Integrated Department of Immunology, University of Colorado and National Jewish Health, Denver, Colorado, United States of America; 4 Department of Medicine, University of Colorado Denver, Aurora, Colorado, United States of America; Charité, Campus Benjamin Franklin, Germany

## Abstract

The common, co-segregating Toll-like receptor 4 (TLR4) non-synonymous single nucleotide polymorphisms (SNPs), Asp299Gly and Thr399Ile, are associated with hyporesponsiveness to inhaled lipopolysaccharide (LPS) and increased susceptibility to Gram negative pathogens in humans. The purpose of this study was to identify the relative contributions of the Asp299Gly and the Thr399Ile variants in inhibiting the function of TLR4. 293/hMD2-CD14 cell line was transfected with lentiviral constructs containing human wild type (WT) TLR4-EGFP or TLR4-EGFP with Asp299Gly, Thr399Ile or Asp299Gly/Thr399Ile complementary DNA (cDNA). Multiple stable cell lines were established for each construct: three for WT TLR4, Asp299Gly, and Thr399Ile, and only two for Asp299Gly/Thr399Ile mutants and EGFP control. We did not observe a significant effect of polymorphisms on cell surface and intracellular TLR4 expression nor were there any significant differences in TLR4 and EGFP protein levels assessed by Western blotting and confocal microscopy among the multiple cell lines of each of the constructs. All cell lines had a dose-dependent responsiveness to LPS stimulation. However, compared to the WT TLR4, cells expressing TLR4 with Asp299Gly but not Thr399Ile polymorphism produced significantly less (*P*<0.05) IL-8 following LPS stimulation. Similarly, cells expressing TLR4 Asp299Gly but not Thr399Ile allele had significantly lower percentage of phosphorylated and total NF-κB P65 following LPS stimulation. While we could not do statistics on the Asp299Gly/Thr399Ile group, we observed a reduced responsiveness to LPS compared to WT TLR4. Taken together, we observed that the TLR4 Asp299Gly variant, but not the Thr399Ile variant, is responsible for impaired responsiveness of TLR4 to LPS and corresponding activation of NF-κB.

## Introduction

Among the TLR family, TLR4 has been shown to recognize structurally unrelated PAMPs including Gram-negative enterobacterial LPS [1]–[3], respiratory syncytial virus (RSV) fusion (F) protein [4], and chlamydial heat shock protein (Hsp) 60 [5]. In addition, endogenous molecules such as fibrinogen [6], [7], fibronectin [8], hyaluronan [9], [10], and surfactant protein A [11], [12] also signal through TLR4. TLR4 signaling involves the adaptor molecules MyD88 and TRIF. Signaling through the MyD88 adaptor leads to early NF-κB activation and pro-inflammatory cytokine production while signaling through the TRIF adaptor gives rise to late NF-κB activation and production of type I interferons as well as other cytokines.

Structural analysis of TLR4 demonstrated that the receptor consists of three domains: an extracellular leucine-rich-repeat (LRR) domain, a transmembrane domain, and an intracellular Toll/interleukin-1 receptor (TIR) domain [13]. TLR4 does not recognize and bind to LPS directly but needs three accessory molecules: LPS-binding protein (LBP), Myeloid differentiation factor 2 (MD-2), and CD14. LBP binds the lipid A moiety of LPS and transfers LPS monomers to soluble or membrane bound CD14 [14]–[17] which in turn transfers LPS to secreted MD-2. Association of the MD-2:LPS complex to the TLR4 ectodomain finally triggers MD-2 and TLR4 conformational changes, resulting in the oligomerization of the TLR4 TIR domain and initiation of downstream signaling transduction [18].

Our group was first to identify two non-synonymous *TLR4* SNPs (Asp299Gly and Thr399Ile) at frequencies up to 10% [19] and commonly co-segregating in European Caucasian but not in African populations [20]. Both of the TLR4 SNPs confer an alteration to the extracellular domain of the TLR4 receptor. It has been demonstrated that the two SNPs, especially the Asp299Gly SNP, are associated with hyporesponsiveness to inhaled LPS but increased susceptibility to Gram negative pathogens in humans [21]–[24]and a decreased risk of atherosclerosis [25].

The molecular mechanisms involved in the diminished LPS responsiveness of individuals expressing the Asp299Gly and Thr399Ile TLR4 polymorphisms have not been fully elucidated. Some published studies have shown changes in cell surface expression of TLR4 as a consequence of the polymorphisms [22], [26] however, this is not consistently observed [27]. Recent crystal structure of the human TLR4 (Asp299Gly/Thr399Ile)·MD-2·LPS complex showed that the tertiary complex is similar to that of the human wild type TLR4·MD-2·LPS complex but it appears that local structural differences might affect the binding of ligands in the region around Asp299Gly, but not Thr399Ile [28].

The aim of this study was to elucidate the mechanism(s) of Asp299Gly- and/or Thr399Ile–mediated inhibition of TLR4 function. Unlike most of previous publications that used transient transfections to study TLR4 WT and polymorphic mRNA and protein expression [28]–[30], we established stable 293/hMD2-CD14 cell lines transfected with a lentiviral construct containing human wild type TLR4-EGFP, and TLR4-EGFP with Asp299Gly, Thr399Ile or Asp299Gly/Thr399Ile complementary DNA (cDNA). We demonstrated that TLR4 Asp299Gly but not Thre399Ile polymorphism led to an impaired responsiveness of TLR4 to LPS and the corresponding activation of NF-κB.

## Materials and Methods

### Reagents and Instruments

pLenti4/TLR4-WT-flag-tagged/TO/V5-Dest vector was a generous gift from Prof. Scott Friedman (Mount Sinai School of Medicine, New York). QuikchangeII-E site-directed mutagenesis kit was purchased from Agilent Technologies. Nhe I-HF and BamH I-HF restriction enzymes were purchased from New England Biolabs. pEGFP-n1 vector was purchased from Clontech. pCR8/GW/TOPO entrez vector, LR recombination reaction kit, ViraPower Packaging Mix, 293FT cell line, Opti-MEM I Medium, Lipofectamine2000, Zeocin, APEX Alexa Fluor 647 Antibody Labeling Kit, and CellTracker probe were purchased from Invitrogen/LifeTechnologies. 293/hMD2-CD14 cell line was purchased from Invivogen, USA. The following antibodies were used: mouse anti-human TLR4-APC antibody, mouse anti-human TLR4 purified antibody, rat IgG2a K isotype control APC, mouse IgG1 isotype control Alexa Fluor647 and mouse IgG2b isotype control Alexa Fluor647 (eBioscience); rat anti-mouse CD16/CD32, anti-NF-κB p65 antibody (BD Biosciences); PerCP anti-human IL-8 and PerCP mouse Ig G2a isotype control (Biolegend); horseradish peroxidase-conjugated goat anti-rabbit IgG (Invitrogen/LifeTechnologies); rabbit polyclonal antibody against human TLR4 (Santa Cruz Biotechnology, Inc); and rabbit monoclonal antibody against human β-actin (Cell Signaling). LPS from *E. coli* O111:B4 was obtained from LIST Biological Laboratories. Additional reagents used were the following: Human CXCL8/IL-8 DuoSet ELISA (R&D Systems), PhosSTOP (Roche Applied Science), Bradford protein assay (Bio-Rad), and Chemiluminescence detection solution (General BioSciences).

Sequencing was performed on the 3730xl DNA analyzer and genotyping on the 7900HT Fast Real-Time PCR System (Applied Biosystems). Flow cytometry was performed on the FACScan flow cytometer (Becton-Dickinson), laser scanning confocal microscopy on the Zeiss LSM 510 (Zeiss Corporation), and ELISA plates were read on the Synergy HT Multi-Mode Plate Reader (Bio-Tek).

### Vector Construction

Vectors were constructed using standard approaches [31] with some modifications. TLR4 Asp299Gly, Thr399Ile or Asp299Gly/Thr399Ile SNPs were generated by a Quikchange II-E site-directed mutagenesis kit on the original pLenti4/TLR4-WT-flag-tagged/TO/V5-Dest construct containing a full length hu-TLR4 cDNA with the TLR4-WT allele. An EGFP sequence from pEGFP-n1 was cloned at the C-terminus of the TLR4 WT and mutant cDNA using Nhe I-HF and BamH I-HF restriction sites. The products were then TOPO-cloned into pCR8/GW/TOPO entrez vector and further transferred into destination vectors via LR recombination reactions. The vector sequences were validated by sequencing.

### Lentivirus Generation

Replication-incompetent lentiviruses were prepared by co-transfecting several plasmids into 293FT cells according to manufacturer's instruction. Briefly, 5.0×10^6^ 293FT cells were seeded in a 10 cm tissue culture dish without antibiotics of DMEM containing 10% FBS, 4 mM L-glutamine, 0.1 mM MEM Non-Essential Amino Acids the day before transfection (Day 1). After overnight incubation (Day 2), cells were co-transfected with 9 μg of ViraPower Packaging Mix and 3 μg of EGFP-tagged TLR4 WT or SNP-expressing pLenti4/TO/V5-GW constructs or a pLenti4/TO/V5-GW/EGFP control plasmid in Opti-MEM I Medium using Lipofectamine2000 mediated transfection. After another 24 hours (Day 3), supernatants were changed to 10 ml fresh complete media. Viral supernatants were harvested at 48 hours and 72 hours following transfections, centrifuged and filtered and pipetted into cryovials in 1 ml aliquots and frozen down at −80°C. The titers of the viruses ranged from 5×10^5^ to 10×10^5^ transducing units (TU)/ml.

### Reconstitution of TLR4 WT/SNPs-EGFP Expression in the 293/hMD2-CD14 Cell Line

The 293/hMD2-CD14 cell line was transfected with lentiviral constructs containing human TLR4-EGFP with WT, Asp299Gly, Thr399Ile, or Asp299Gly/Thr399Ile alleles, or EGFP cDNA in the presence of hexadimethrine bromide at the MOI (multiplicity of infection) of 1. The infected cells were selected and maintained with complete media containing 100 μg/ml Zeocin. After 8–10 weeks of selection, multiple cell lines were established for each genotype group: three TLR4 WT (lines 8, 10, 11, referred to as WT-8, 10, 11), three Asp299Gly (lines 1, 4, 6; D299G-1, 4, 6), three Thr399Ile (lines 4, 11, 13; T399I-4, 11, 13), two Asp299Gly/Thr399Ile (lines 1, 3; D299G/T399I-1, 3) and two EGFP (lines 1, 2) control stable cell lines. All cell lines were genotyped by TaqMan SNP genotyping assay.

### Cell Proliferation Rates

Cell proliferation rates of each cell line were measured using CellTracker probe according to manufacturer's directions.

### Detection of Cell Surface, Intracellular and Total TLR4 Expression by Flow Cytometry

Cell surface, intracellular and total TLR4 expression levels of each cell line were detected by flow cytometry using mouse anti-human TLR4-APC antibody. Briefly, 3×10^6^ cells of each cell line were fixed with 1% formaldehyde. For the total TLR4 staining, cells were incubated with mouse anti-human TLR4-APC antibody (1∶50 dilution) and rat anti-mouse CD16/CD32 (Mouse BD Fc Block, 1∶100 dilution) on ice for 30 minutes before and after permeabilization using BD Cytofix/Cytoperm Fixation/Permeabilization Solution Kit. For cell surface staining, cells were stained without permeabilization. For intracellular staining, cells were incubated with mouse anti-human TLR4 purified antibody (1∶50 dilution) and rat anti-mouse CD16/CD32 (1∶100 dilution) on ice for 30 minutes before permeabilization to block the surface TLR4. After permeabilization, cells were incubated with mouse anti-human TLR4-APC antibody (1∶50 dilution) and rat anti-mouse CD16/CD32 (1∶100 dilution) on ice for 30 minutes. Each sample was tested on FACSan flow cytometer (CellQuest Pro Version 5.2). 1∶100 dilution of rat IgG2a K isotype control APC was used for isotype control. All experiments were repeated at least 3 times.

### TLR4-EGFP Expression by Confocal Microscopy

Cell lines were seeded (5×10^3^ cells) on poly-L-lysine-coated 4-well Lab-Tek Chambered Coverglass in complete media with 100 μg/L Zeocin and cultured for 20 hours followed by gentle washing with cold PBS. Images were acquired using laser scanning confocal microscope at excitation wavelength 488 nm and standard emission filter for GFP detection (TLR4-EGFP).

### LPS stimulation, Cytokine Production, and NF-κB Activation

Concentrations of secreted IL-8 in the supernatants after LPS stimulation were measured by ELISA. Briefly, 3×10^5^ cells of each cell line were seeded in 24 well plates in complete media containing 100 μg/ml Zeocin. After 16–20 hours incubation, cells were stimulated with 0, 0.1, 0.5, 2 and 20 ng/L LPS for 24 hours. Concentrations of human IL-8 were measured by Human CXCL8/IL-8 DuoSet ELISA kit. Each experiment was repeated three times.

Intracellular concentrations of human IL-8, phosphorylated and total NF-κB p65 after LPS stimulation were also determined by intracellular flow cytometry analysis. Briefly, 16–20 hours before stimulation, 1.0×10^6^ cells of each cell line were seeded in 6-well plates in complete media containing 100 μg/ml Zeocin. For intracellular human IL-8 detection, cells were stimulated with 20 ng/ml LPS 18 hours, followed by treatment with 2 μl of GolgiPlug for another 6 hours, and cells were collected for routine flow cytometry preparation. For NF-κB phosphorylated p65 detection, cells were stimulated with 10 ng/ml LPS for 0 min, 30 min, 60 min and 120 min and then cells were collected for flow cytometry staining. Intracellular human IL-8, phosphorylated and total NF-κB p65 flow staining were measured respectively by 1: 50 dilution of PerCP anti-human IL-8, phosphorylated NF-κB p65 Alexa Fluor 647and total anti- NF-κB p65 antibody with Alexa Fluor 647 labeled according to manufacturer's instructions. Fold changes of percentages or MFI of IL-8, phosphorylated NF-κB p65 and total NF-κB p65 positive cells were used to compare responsiveness of cell lines to LPS stimulations compared with each cell line's baseline condition with no LPS stimulations (referred to as fold increases) [31]. Considering that LPS responsiveness may include not only percentage of positive cells but also MFI, we used fold changes of percentages multiplied with MFI to compare their responsiveness as well. Each experiment was repeated at least three times.

### Western Blotting

5×10^6^ cells from each cell line were harvested, lysed and centrifuged, and the protein concentrations were detected by Bradford protein assay. 50 μg of protein samples were subjected to SDS-PAGE and transferred to PVDF membrane, blocked with 5% non-fat milk in TBST-0.1% Tween 20 and incubated with rabbit polyclonal antibody against human TLR4 and rabbit monoclonal antibody against human β-actin, followed by horseradish peroxidase-conjugated goat anti-rabbit IgG. Blots were visualized using chemiluminescence detection. Semi-quantitative analysis for the expression levels of proteins was performed by densitometry.

### Statistical Analysis

All results are expressed as the mean ± standard error of the mean (SEM). *P* values (one way ANOVA, two-tailed t-test, and Newman-Keuls multiple comparison) of at least three independent determinations were calculated with GraphPad Prism 5. As there were just two TLR4 Asp299Gly/Thr399Ile cell lines established, we could not do statistical test on this group. Data were considered to be statistically significant at *P*<0.05. Flowjo 7.6.5 software was used for flow cytometry data analysis.

## Results

### Generation of Cell Lines Expressing Lentiviral Vectors with Full Length TLR4 WT/SNPs-EGFP cDNA

We generated 13 stable cell lines expressing 5 lentiviral vectors (EGFP control, TLR4-WT-EGFP, TLR4-Asp299Gly-EGFP, TLR4-Thr399Ile-EGFP and TLR4-Asp299Gly/Thr399Ile-EGFP) for the purpose of assessing the effect of TLR4 polymorphisms Asp299Gly and Thr399Ile on TLR4 expression and function (Table 1). Inserts were confirmed by both Sanger sequencing and Taqman genotyping of the TLR4 Asp299Gly and Thre399Ile polymorphisms. We tested proliferation rates of the established cell lines and demonstrated no differences in proliferation among TLR4 genotypic groups (Figure S1).

**Table 1:** Lentiviral vectors and cell lines stably expressing these vectors.

Lenti-vectors	Cell Line (Abbreviated Name)
pLenti4/EGFP/TO/V5-Dest	293/hMD2-CD14-EGFP-1, 2 (EGFP-1, 2)
pLenti4/hu-TLR4-WT-EGFP/TO/V5-Dest	293/hMD2-CD14-TLR4-WT-EGFP-8, 10, 11 (WT-8, 10, 11)
pLenti4/hu-TLR4-Asp299Gly-EGFP/TO/V5-Dest	293/hMD2-CD14-TLR4-Asp299Gly-EGFP-1, 4, 6 (D299G-1, 4, 6)
pLenti4/hu-TLR4-Thr399Ile-EGFP/TO/V5-Dest	293/hMD2-CD14-TLR4-Thr399Ile-EGFP-4, 11, 13 (T399I-11, 13)
pLenti4/hu-TLR4-Asp299Gly/Thr399Ile-EGFP/TO/V5-Dest	293/hMD2-CD14-TLR4- Asp299Gly/Thr399Ile -EGFP-1, 3 (D299G/T399I-1, 3)

Note: In Table 1, each cell line was named by different genotypes and numbers. D299G, T399I and D299G/T399I represented cell lines of TLR4 Asp299Gly, Thr399Ile and Asp299Gly/Thr399Ile genotypes respectively. Numbers were used when doing cell line selection.

### TLR4 SNPs Have No Effect on TLR4 Protein Expression and Subcellular Distribution

**Figure 1:**
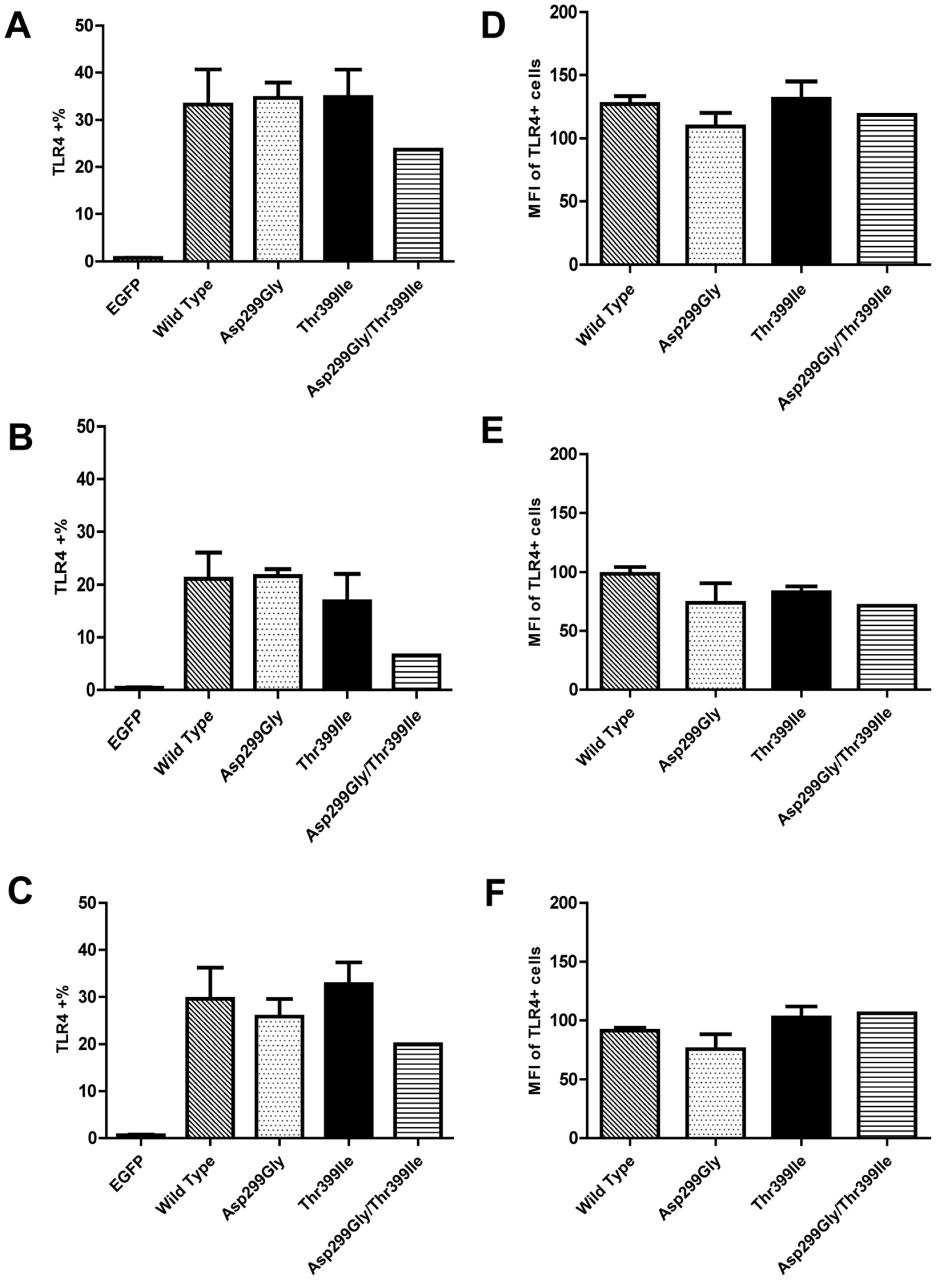
Comparison of total, cell surface and intracellular TLR4 expression by TLR4 genotype group. Each cell line was examined for total (**A, D**), cell surface (**B, E**) and intracellular (**C, F**) TLR4 expression by flow cytometry using the mouse anti-human TLR4-APC antibody. Percentage of total, cell surface and intracellular TLR4 positive cells for cell line groups are shown in panels (**A**–**C**). Displayed in panels (**D**–**F**) are mean fluorescent intensities (MFI) of TLR4 positive cells in each genotype group. All data are presented as mean ± SEM of three independent experiments. Statistical comparisons were performed using One-way ANOVA and Newman-Keuls post-hoc test among the TLR4 genotype groups (n = 3) except the Asp299Gly/Thr399Ile cell group (n = 2).

To explore TLR4 expression and distribution, we examined total, cell surface, and intracellular TLR4 expression by flow cytometry analysis (Figure 1 and Figure S2). We observed neither differences in the percentage of TLR4-positive cells nor mean TLR4 expression (measured by MFI) of TLR4-positive cells in the EGFP-positive cell population based on genotype (n = 3, *P*>0.05) (Figure 1A and 1D). Similarly, TLR4 genotype did not influence the percentage of TLR4-positive cells with surface (Figure 1B) or intracellular expression (Figure 1C) of the receptor nor mean surface (Figure 1E) or intracellular (Figure 1F) TLR4 expression. In Figure 1B, fewer of the Asp299Gly/Thre399Ile cells expressed TLR4 on the surface (6.55%±1.49%) than those of the wild type (21.13%±8.44) but we could not test for significance due to the fact that there are only two cell lines in the Asp299Gly/Thre399Ile mutant group.

**Figure 2:**
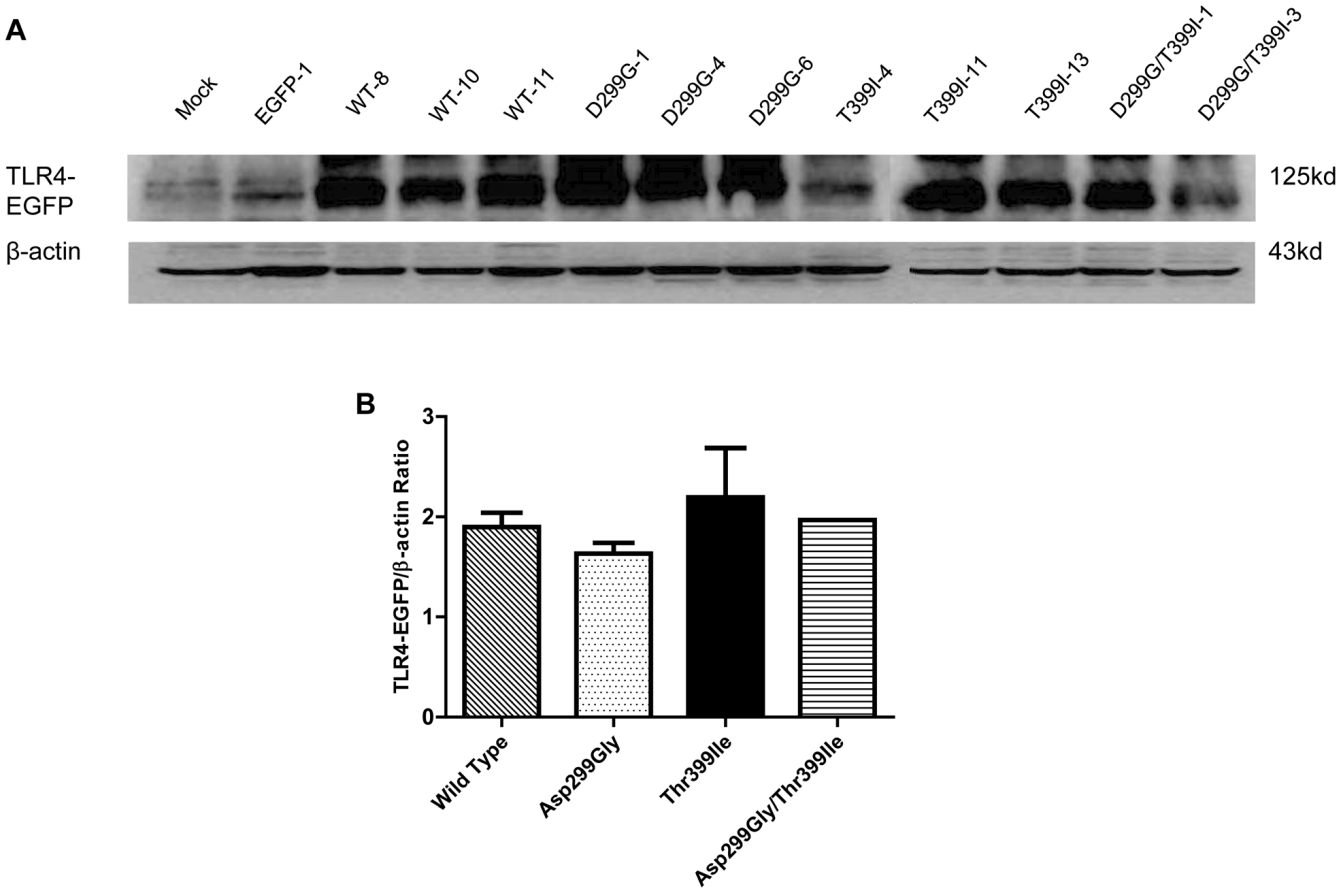
Western blot and quantitative densitometry analysis of TLR4-EGFP and β-actin protein expression. Western blot and quantitative densitometry analysis of TLR4-EGFP and β-actin protein expression in each cell line (**A**) and genotype group (**B**). Plotted in panel *B* are mean TLR4-EGFP/β-actin ratios of densitometry for each genotype cell group. Statistical comparisons were performed using One-way ANOVA and Newman-Keuls post-hoc test among the TLR4 genotype groups (n = 3) except the Asp299Gly/Thr399Ile cell group.

**Figure 3:**
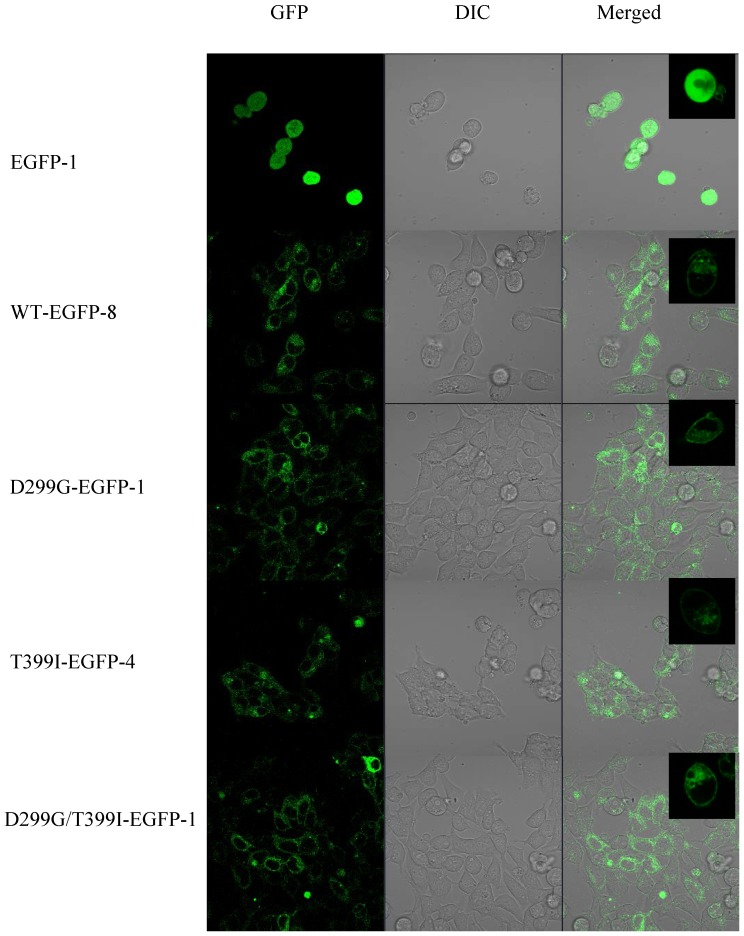
Confocal microscopy analysis of wild type and polymorphic TLR4-EGFP protein expression levels. GFP fluorescence, differential interference contrast (DIC) and merged images of 293/Hu-MD2-CD14 cell lines with human WT or polymorphic TLR4-EGFP protein and EGFP control were taken at 400X magnification simultaneously. One representative cell line for each genotype group is displayed. Other cell lines in each group had very similar patterns of TLR4-EGFP expression.

We also performed TLR4-EGFP Western blotting to further compare TLR4 protein expression levels in multiple cell lines of each TLR4 genotype (Figure 2). Densitometry analysis demonstrated that ratios of TLR4-EGFR/β-actin had no significant difference among cell lines with wild type and polymorphic TLR4 (Figure 2B). Finally, confocal images of each cell line demonstrated that EGFP expression and distributions were comparable among the genotypic groups (Figure 3). Taken together, our data demonstrate that TLR4 Asp299Gly and Thre399Ile SNPs have no impact on total expression nor subcellular localization of the TLR4 protein. The effect of the Asp299Gly/Thre399Ile SNP is similar to the TLR4 Asp299Gly and Thre399Ile SNPs but more than two cell lines would be needed to test for significance.

### TLR4 Asp299Gly but Not Thr399Ile Polymorphism Blunts Response to LPS Stimulation

**Figure 4:**
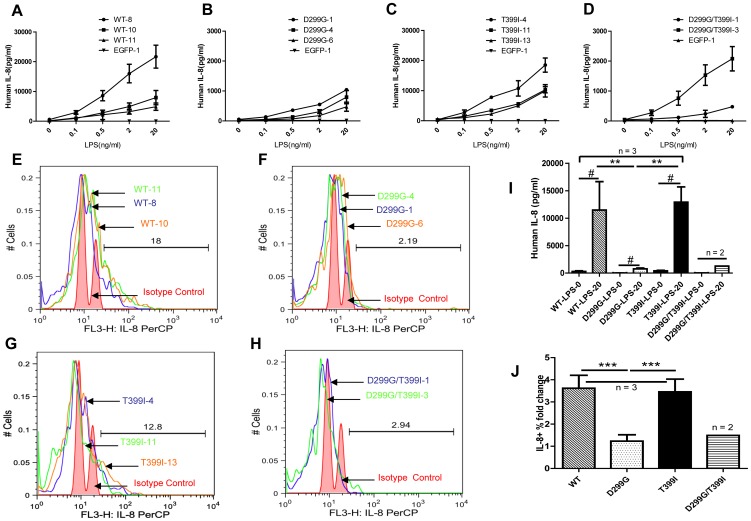
The effect of TLR4 SNPs on cytokine production in response to LPS. Shown in panels (**A**–**D**) are comparisons of dose-response curves to LPS stimulations of cell lines in each TLR4 genotype group. 3.0×10^5^ cells were stimulated for 24 hours with a series of LPS concentrations and conditioned media from each cell line was tested for human IL-8 secretion by ELISA. (I) Human IL-8 production 24 hours after LPS treatment for different genotype cell groups. WT-LPS-0 and WT-LPS-20 represent TLR4 wild type cell lines stimulated with 0 and 20 ng/ml LPS (same annotation for other cell groups). (**E**–**H**) Percentage of TLR4- and EGFP-positive cells that are also positive for IL-8 as assessed by flow cytometry analysis. (**J**) Fold change (percentage multiplied by MFI) of intracellular human IL-8 positive cells after LPS(20 ng/ml)stimulation for the four genotype groups. # no LPS stimulation vs 20 ng/ml LPS stimulation within the same genotypic cell group, *P*<0.05; ** and *** vs Asp299Gly group, *p*<0.01 (I) and 0.001(J). Statistical comparisons were performed using One-way ANOVA and Newman-Keuls post-hoc test among the TLR4 genotype groups (n = 3) except the Asp299Gly/Thr399Ile cell group (n = 2).

To elucidate consequences of the Asp299Gly and Thr399Gly polymorphisms on TLR4 function, we performed dose-response tests of 293/hMD2-CD14 cells lines with lentiviral constructs to increasing concentrations of LPS. IL-8 concentrations in the supernatant measured by ELISA showed dose-dependent responsiveness to LPS stimulations of all 293/hMD2-CD14 cell lines (Figure 4A–D). Cells expressing TLR4 Asp299Gly produced less IL-8 than cells expressing TLR4 wild type and TLR4 with the Thr399Ile mutation (Figure 4A–D and 4I). (*P*<0.01) (Figure 4I). We also used flow cytometry to compare the percentages of intracellular IL-8-positive cells (Figure 4E–H) and demonstrated that TLR4 wild type and Thr399Ile cell groups had (3.61±0.59) and (3.46±0.58) fold increase, respectively, of the percentage of cells producing IL-8 in response to 20 ng/ml LPS stimulation whereas the TLR4 Asp299Gly had only (1.23±0.29) fold increase (n = 3, *P*<0.01) (Figure 4J). TLR4 Asp299Gly/Thre399Ile cell lines had similar LPS responsiveness to that of TLR4 Asp299Gly but more cell lines would be needed to confirm. Taken together, these results demonstrate the effect of the Asp299Gly but not Thr399Ile mutation on TLR4 responsiveness to LPS as measured by cytokine production.

### TLR4 Asp299Gly but Not Thr399Ile Polymorphism Reduces NF-κB Signaling

**Figure 5:**
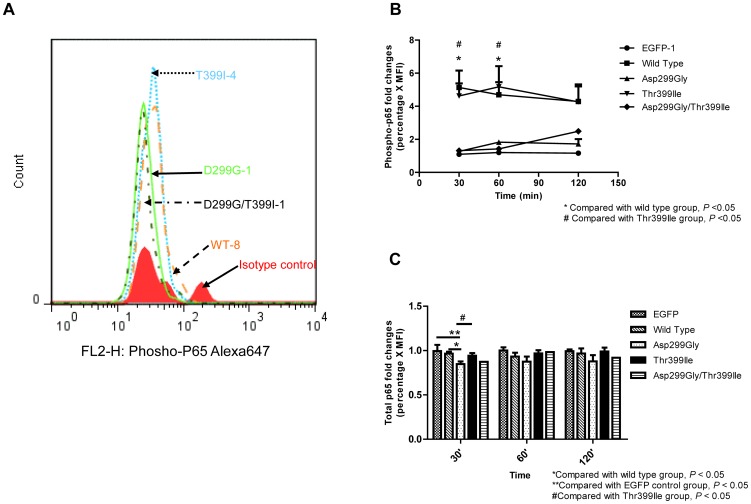
The impact of TLR4 SNPs on LPS-induced NF-κB signaling. (**A**) Overlay of percentages of NF-κB phospho-p65 positive cells of one representative cell line in each genotype group after 30 min LPS (10ng/ml) stimulation. Fold changes (percentage of cells multiplied with MFI) of intracellular phosphorylated (**B**) and total (**C**) p65 NF-κB positive cells of different genotype cell groups following 0 min, 30 min, 60 min, and 120 min LPS(10ng/ml)stimulation. Statistical comparisons were performed using One-way ANOVA and Newman-Keuls post-hoc test among the TLR4 genotype groups (n = 3) except the Asp299Gly/Thr399Ile cell group (n = 2).

To further explore the mechanism of blunted response of TLR4 with the Asp299Gly mutation to LPS, we examined the NF-κB signaling pathway by detecting phosphorylated and total p65 NF-κB subunit in response to 10 ng/ml LPS by flow cytometry analysis (Figure 5). This analysis was done for one representative cell line for each genotype group. Pospho-p65 was detected in lower amounts in cell lines expressing TLR4 with Asp299Gly and Asp299Gly/Thr399Ile mutations than in WT TLR4 or TLR4 with the Thr399Ile mutation alone (Figure 5A). Time course experiments (Figure 5B) demonstrated a significant reduction in fold change (percentage of cells multiplied with MFI) [31] of phospho-p65 induction in response to LPS stimulation in the Asp299Gly compared to wild type and Thr399Ile groups at 30–60 min with differences diminishing at 120 min (Asp299Gly/Thr399Ile TLR4 response similar to Asp299Gly), suggesting the effect of the Asp299Gly mutation on response to LPS in these cell lines. In addition to phospo-p65, we also examined the amount of total p65 NF-κB in these same cell lines (Figure 5C). Flow cytometry results demonstrated significantly lower fold changes (percentage of positive cells multiplied with MFI) of total p65 NF-κB positive cells after 30 min in the Asp299Gly (0.85±0.03) group compared with the wild type (0.97±0.02) (n = 3, *P*<0.05), while no significant change was observed in Thr399Ile cells (0.97±0.04) compared to WT (n = 3, *P*>0.05).The Asp299Gly/Thr399Ile mutation had a similar effect to the Asp299Gly. There was no significant difference in total p65 NF-κB fold change at 60 min and 120 min post-LPS among the groups (n = 3, *P*>0.05). Taken together, TLR4 Asp299Gly but not Thr399Ile polymorphism reduced but also delayed TLR4 response to LPS stimulation as measured by NF-κB p65 subunit phosphorylation and total NF-κB p65.

## Discussion

Our results demonstrate, using stably transfected cell lines, that the TLR4 Asp299Gly and Thr399Ile polymorphisms do not influence TLR4 protein expression and subcellular localization. However, the Asp299Gly, but not Thr399Ile, polymorphism blunts TLR4 function, as assessed by cytokine production and NF-κB stimulation in response to LPS.

It is established that the intensity of LPS responsiveness is partly regulated by the levels of TLR4 present of the cell surface [32]. Our extensive analysis by flow cytometry, Western blotting and confocal microscopy demonstrated no difference in TLR4 protein expression or cellular localization as a function of the presence of Asp299Gly and Thr399Ile polymorphisms. The double-mutant demonstrated no effect on TLR4 protein expression or cellular localization either except in Figure 1B, where it appeared that TLR4 surface expression was lower among the double-mutant cell group than the other groups; however, we could not test for significance due to small number of cell lines in the double-mutant group. These results are consistent with some of the previous studies [27], [33]. Rallabhandi et al. [27] examined TLR4 mRNA and protein expression in HEK293T cell lines transiently co-transfected with wild type or mutant TLR4, MD-2 and CD14 and found no significant difference in TLR4 expression among the wild type or mutant TLR4 cell lines. Lundberg et al. [33] explored TLR4 surface expression in CD14+ PBMC of subjects that are wild type or polymorphic for the Asp299Gly SNP and also detected no differences. Nevertheless, other studies detected differences in surface expression of TLR4 based on the genotype [22], [26]. Using a polyclonal anti-TLR4 antibody, we previously [22] found reduced surface expression of TLR4 in human airway epithelia from TLR4 Asp299Gly/Thr399Ile individuals compared to that of wild type individuals. Tulic et al. [26] reported that individuals heterozygote for TLR4 Asp299Gly or Thr399Ile had lower surface TLR4 expression on CD14+ PBMCs. One possible explanation for this discrepancy in findings is the presence of MD-2. In studies that co-express TLR4 with MD-2, TLR4 surface expression is not influenced by either of the TLR4 SNPs [[31], [33], [34] and our present experiment]; on the other hand, TLR4 surface expression was influenced by the TLR4 SNPs in the absence of MD-2 [22], [26].

We demonstrate that the presence of the TLR4 Asp299Gly but not Thr399Ile polymorphism leads to impaired TLR4 signaling in response to LPS. Both secreted and intracellular IL-8 were reduced in TLR4 Asp299Gly cell lines in response to LPS compared to that of the wild type and Thr399Ile cell groups. Our results were consistent with previous *in vitro* [22], [26], [27], [34] and *ex vivo* studies [29], [30], [35]–[37]. We also showed reduced protein levels of total and phosphorylated NF-κB as a result of the Asp299Gly but not Thr399Ile SNP at early time points (30–60 min) but not later time points (120 min). These findings are in line with a study that showed diminished IκBα phosphorylation at 30 min and 60 min LPS stimulations but not 120 min in PBMCs from subjects heterozygous for TLR4 Asp299Gly or Thr399Ile patients compared to TLR4 wild type patients [26]. Furthermore, our data confirmed a recent study demonstrating that TLR4 Asp299Gly compromised recruitment of MyD88 and TIR-domain-containing adapter-inducing interferon-β (TRIF) to TLR4 without affecting TLR4 expression, TLR4-MD-2 and LPS binding [38].

Our results were contradicted with an *ex vivo* study in which Ferwerda et al. [20] showed that cells from individuals of African descent carrying only the Asp299Gly variant displayed an increased responsiveness to LPS. They suggested that this hyperresponsiveness may have been an advantage in fighting off malaria, and that this may be the reason for this variant still being present in Africa where malaria is still prevalent, while it has been lost in the European population. While this is a feasible possibility, other technical differences (transfected cells vs PBMCs) may also explain differences in responsiveness Regardless, TLR4 Asp299Gly gene polymorphism and the bacterial origin of LPS should be considered when environmental LPS exposure is evaluated in disease risk or protection [26].

We also note that there is substantial variation within the same polymorphic cell groups. We used qPCR to check insert numbers and they were comparable (data not shown) in all cell lines. As lentiviral-induced transfection was used in this experiment, desired genes (WT and polymorphicTLR4) were integrated into the whole genome randomly. Positions where the TLR4 cDNA inserted may influence TLR4's mRNA expression. We examined mRNA expressions of each cell line (data not shown) and found there was a slight difference within each polymorphic cell group but the differences were not significant (P>0.05).

Taken together, our results indicate that reduced LPS responsiveness of the TLR4 protein mutant for the Asp299Gly allele is not due to diminished surface TLR4 protein expression but that the polymorphism must alter the ability of TLR4 to interact with MD-2 and LPS and/or signal eliciting. An earlier study reported significant differences in NF-κB activation in response to LPS under conditions where WT and polymorphic variants were comparably expressed [27]. In this study, overexpression of Flag-tagged WT and mutant vectors at input levels resulting in agonist-independent signaling led to equivalent NF-κB signaling, suggesting that these mutations in TLR4 affect appropriate interaction with agonist or co-receptor. Crystal structure of the tertiary TLR4·MD-2·LPS complex solved recently for both WT and mutant TLR4 [28] revealed that mutant TLR4 complexes exhibited a similar overall architecture to that of the human wild type TLR4·MD-2·LPS complex but that local structural differences that might affect the binding of the ligands were observed around Asp299Gly, but not around Thr399Ile, SNP site. These protein structure results are consistent with the current findings that the Asp299Gly but not Thr399Ile affects TLR4 signaling. Previous findings on the role of the Thr399Ile mutation were mixed with some demonstrating an intermediate response to LPS [22], [39] and other showing an effect similar to that of the Asp299Gly allele [26], [40]; however, none of these previous studies used a stable transfection system. Our current findings also confirm a recent study and provide a more definitive answer pointing to the role of Asp299Gly, but not the Thr399Ile, in TLR4 function and signaling.

## Supporting Information

Figure S1 **Cell proliferation rates of each cell line were examined by the CellTracker probe according to the manufacturer's directions.** Cells were incubated with the CellTracker probe for 48 hours and then harvested for flow cytometry analysis. Each successive generation in a population is marked by a halving of the cellular fluorescence intensity which is readily followed by flow cytometry. A sum of percentages of all the successive generations of each cell line approximately represents its proliferation rate. These measurements were repeated three times for each cell line and the average with the SEM are shown in the figure. There were no significant differences in cell proliferation rates among the TLR4 genotype groups (P>0.05). Statistical comparisons were performed using One-way ANOVA and Newman-Keuls post-hoc test.
(TIFF)

Figure S2 **Comparison of total, cell surface and intracellular TLR4 expression in each individual cell line.** Cell line was examined for total (A), cell surface (B), and intracellular (C) TLR4 expression by flow cytometry using the mouse anti-human TLR4-APC antibody. Percentages of total, cell surface and intracellular TLR4 positive cells for each cell line are shown. All data are presented as mean ± SEM of three independent experiments. Statistical comparisons were performed using One-way ANOVA and Newman-Keuls post-hoc comparison test.
(TIFF)
