# Supplementary figures and images for: The Toll-Like Receptor 4 Polymorphism Asp299Gly but Not Thr399Ile Influences TLR4 Signaling and Function
Source: PLoS One. 2014 Apr 2;9(4):e93550. doi: 10.1371/journal.pone.0093550 (PMC3973565; doi:10.1371/journal.pone.0093550)

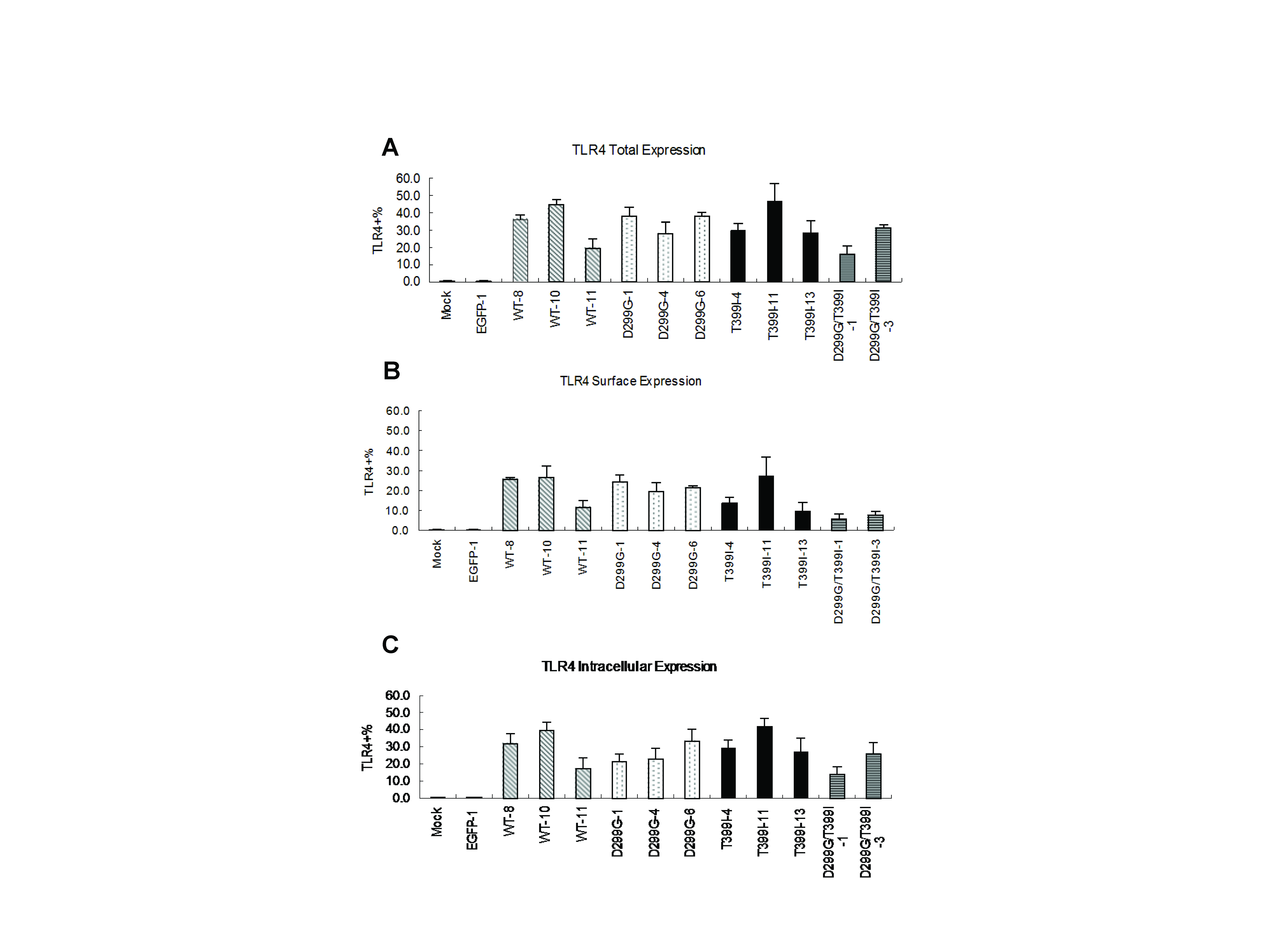

Supplement: Figure S2 — Comparison of total, cell surface and intracellular TLR4 expression in each individual cell line. Cell line was examined for total (A), cell surface (B), and intracellular (C) TLR4 expression by flow cytometry using the mouse anti-human TLR4-APC antibody. Percentages of total, cell surface and intracellular TLR4 positive cells for each cell line are shown. All data are presented as mean ± SEM of three independent experiments. Statistical comparisons were performed using One-way ANOVA and Newman-Keuls post-hoc comparison test. (TIFF) [file pone.0093550.s002.tiff]
